# Histone modifications are specifically relocated during gene activation and nuclear differentiation

**DOI:** 10.1186/1471-2164-10-554

**Published:** 2009-11-24

**Authors:** Katharina Sarah Heyse, Susanne Erika Weber, Hans-Joachim Lipps

**Affiliations:** 1 University Witten/Herdecke, Institute of Cell Biology, Stockumer Str 10, 58453 Witten, Germany

## Abstract

**Background:**

Post-translational histone modifications (PTMs) and their specific distribution on genes play a crucial role in the control of gene expression, but the regulation of their dynamics upon gene activation and differentiation is still poorly understood. Here, we exploit the unique genome organization of ciliates to analyse PTM dynamics during gene activation in the differentiated cell and during nuclear differentiation. In the macronucleus of these cells the DNA is organized into nanochromosomes which represent independent functional units. Therefore, ciliated protozoa represent a simplistic model system to analyse the relevance of histone modifications and their localization for gene expression and differentiation.

**Results:**

We analysed the distribution of three PTMs on six individual nanochromosomes, two of which are silenced in the vegetative cell and only activated during sexual reproduction. We show that a specific relocation of these PTMs correlates with gene activation. Moreover, macronuclear-destined sequences in the differentiating macronucleus display a distribution of PTMs which differs significantly from the PTM patterns of actively transcribed genes.

**Conclusion:**

We show for the first time that a relocation of specific histone modifications takes place during activation of genes. In addition, we demonstrate that genes in a differentiating nucleus are characterised by a specific distribution and composition of PTMs. This allows us to propose a mechanistic model about the relevance of PTMs for gene activation, gene silencing and nuclear differentiation. Results described here will be relevant for eukaryotic cells in general.

## Background

Post-translational modifications (PTMs) of histones, which include methylation, acetylation, phosphorylation and others, play a major role in regulating gene transcription and other nuclear processes [1]. It is known that local chromatin structure and thereby transcriptional regulation is influenced by these PTMs. Moreover, post-translational histone modifications have long-range effects on the overall chromatin structure. While transcriptional activation generally correlates with acetylation of lysines, the function of methylation is more complex, with even mono-, di- or trimethylation at specific sites having different effects on the transcriptional status [2]. Furthermore, as PTMs are interdependent, the nucleosomal context of modifications plays a crucial role in regulating chromatin structure [1]. It has been reported that specific modifications tend to cluster at the 5'- or 3'-ends of genes while others are evenly spread over entire gene domains [3-5]. The importance of histone PTMs is undoubtedly complex, and while many functional correlations are well-established further studies are required to establish in detail how patterns of PTMs differ between actively transcribed and transcriptionally repressed genes and how these patterns change upon activation of a gene or during differentiation [6-8].

The unique genome organization of stichotrichous ciliates, such as *Stylonychia*, provides an attractive biological model system to study changes in PTM patterns during gene activation on individual genes and during a nuclear differentiation process. The macronuclear genome of these cells is fragmented into individual short DNA molecules (nanochromosomes). They are derived after sexual reproduction (conjugation) from a micronuclear derivative in a well characterised differentiation process, which includes extensive DNA-reorganization, DNA-elimination and DNA-fragmentation events [9]. Nanochromosomes are individual functional units, which operate independently of the complex effects of chromosomal context that normally contribute to expression of eukaryotic genomes. Although the rate of transcription is very high in the macronucleus not all nanochromosomes are actively expressed during vegetative growth. Some are only expressed in the course of conjugation [10]. Hence, a detailed analysis of the PTM distribution on nanochromosomes should provide a simplified system to monitor chromatin structure during changes in gene expression.

We have shown before that after sexual reproduction the developing macronucleus (macronuclear anlage) adopts a permissive chromatin structure, characterised by the presence of histone modifications typical for active chromatin. In later stages of differentiation repressive histone modifications are introduced *de novo *and specify DNA sequences to be eliminated while permissive histone modifications stay associated with sequences that will be retained in the macronucleus [11]. This well defined nuclear differentiation allows the analysis of PTM pattern on genes during a differentiation process.

Here we show that H3K14ac, H3K4me3 and H3K4me1 each exhibit a characteristic distribution on nanochromosomes in the differentiated macronucleus. These patterns differ between nanochromosomes expressed during vegetative growth and those expressed only during sexual reproduction. Moreover, characteristic relocation of these PTMs occurs during gene activation. Finally, we demonstrate that in the developing anlage macronuclear-destined sequences (MDS) show specific distributions of PTMs, which differ remarkably from those of actively transcribed genes within the fully differentiated macronucleus. These observations allow us to propose a mechanistic model how PTM patterns correlate with gene activation, gene silencing and nuclear differentiation.

## Results

In the current work we studied the relevance of post-translational histone modifications for gene activation, analysed their dynamics on nanochromosomes specifically activated during sexual reproduction and their fate during a nuclear differentiation process. In our analysis of the gene-wide distribution of histone modifications we selected six macronuclear nanochromosomes of which four, the actin I gene, the DNA polymerase alpha gene, the histone H4 gene and a 1.1 kb gene of unknown function are transcribed during vegetative growth and two, mdp1 and mdp2, are silenced in the vegetative macronucleus and become expressed exclusively during conjugation [10].

**Figure 1:**
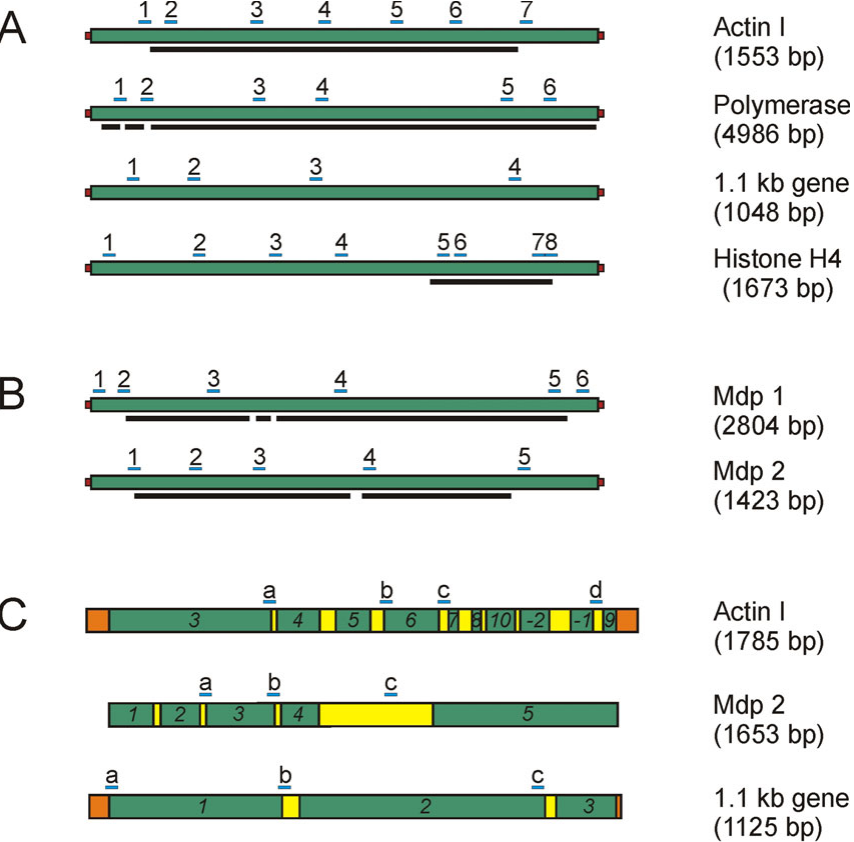
**Schematic illustration of macronuclear nanochromosomes and their micronuclear sequences**. (A) Macronuclear nanochromosomes, transcribed during vegetative growth. (B) Macronuclear nanochromosomes, silenced during vegetative growth. (A,B) Total length of nanochromosome is indicated in brackets. DNA sequences are shown in green, telomeres are red. Black bars below nanochromosomes indicate the coding region of each gene, partly interrupted by introns and flanked by non-coding sequences. Blue bars above nanochromosomes show the positions of qRT-PCR fragments. Numbers show the order of PCR fragments, for primer sequences see Additional file 5. (C) Micronuclear sequences of the actin I, mdp2 and the 1.1 kb gene. Macronuclear-destined sequences (MDS) in green are interrupted by micronucleus-specific internal eliminated sequences (IES) in yellow and partly flanked by further micronucleus-specific sequences (orange). MDSs of the actin I gene are found in a scrambled disorder in micronuclei and anlagen (3-4-5-6-7-8-10-(-2)-(-1)-9) and are reorganized into the correct order (1-2-3-4-5-6-7-8-9-10) upon differentiation [19]. Total length of sequence is indicated in brackets. Blue bars above DNA sequence indicate positions of qRT-PCR fragments. Letters show order of PCR fragments, for corresponding primer sequences see Additional file 6.

These nanochromosomes show significant differences in the length of sequences flanking the open reading frame (Figure 1A, B). While the actin I nanochromosome [GenBank accession number DQ108617] and the nanochromosome encoding the DNA polymerase alpha gene [GenBank accession number AF194338] have short flanking sequences, the nanochromosome encoding a histone H4 gene [GenBank accession number X16018] was chosen because of its unusually long 5'-flanking region. Neither the protein encoded on the 1.1 kb nanochromosome [pCE7, GenBank accession number X72958] nor its precise open reading frame are characterised, therefore the lengths of the flanking sequences are unknown. For analysis of conjugation-specific gene activation the genes encoding mdp1 and mdp2 were chosen. Mdp1 [GenBank accession number AY261996] encodes a PIWI-homologue [10], a protein which is a member of the RNAi-pathway in many organisms and is known to play a crucial role in the course of conjugation in ciliates. The protein expressed by mdp2 [GenBank accession number AY261997] is not known. The PTM pattern on the mdp1 and mdp2 nanochromosomes was studied both in their silenced form in vegetative cells and after activation during conjugation. For further details on sequences see Additional file 1. Finally, we studied histone modifications on genes in the differentiating macronucleus after conjugation. We analysed the PTM patterns on the actin I [GenBank accession number DQ108616], mdp2 [GenBank accession number GU111958] and the 1.1 kb gene [GenBank accession number X72958, pCE7] sequences in the macronuclear anlage (Figure 1C) in the early polytene chromosome stage (30 hours post conjugation).

Histone modifications shown to be present in macronuclei and associated with macronuclear-specific DNA sequences in the developing macronucleus are H3K14ac, H3K4me3 und H3K4me1 [11] and therefore used in this study. Histone acetylations are very dynamic and generally correlate with an open chromatin state and active transcription [1]. High-resolution analysis revealed that H3K14ac accumulates predominantly at promoter regions and transcriptional start sites (TSSs) [4,5,12], in many cases co-localizing with H3K4me3 [4]. Likewise, trimethylated H3K4 is associated with actively transcribed genes and decorates their promoters as well as the 5'-ends of coding regions [13,14]. Although H3K14ac and H3K4me3 are known to be generally associated with actively transcribed genes, these modifications have also been found to decorate repressed genes in humans [15,16].

To date, little is known about the role of H3K4me1 in the regulation of gene expression. H3K4me1 is found to accumulate towards the 3'-end of genes and is commonly linked to active transcription [15,17].

### The PTM patterns of actively transcribed genes

**Figure 2:**
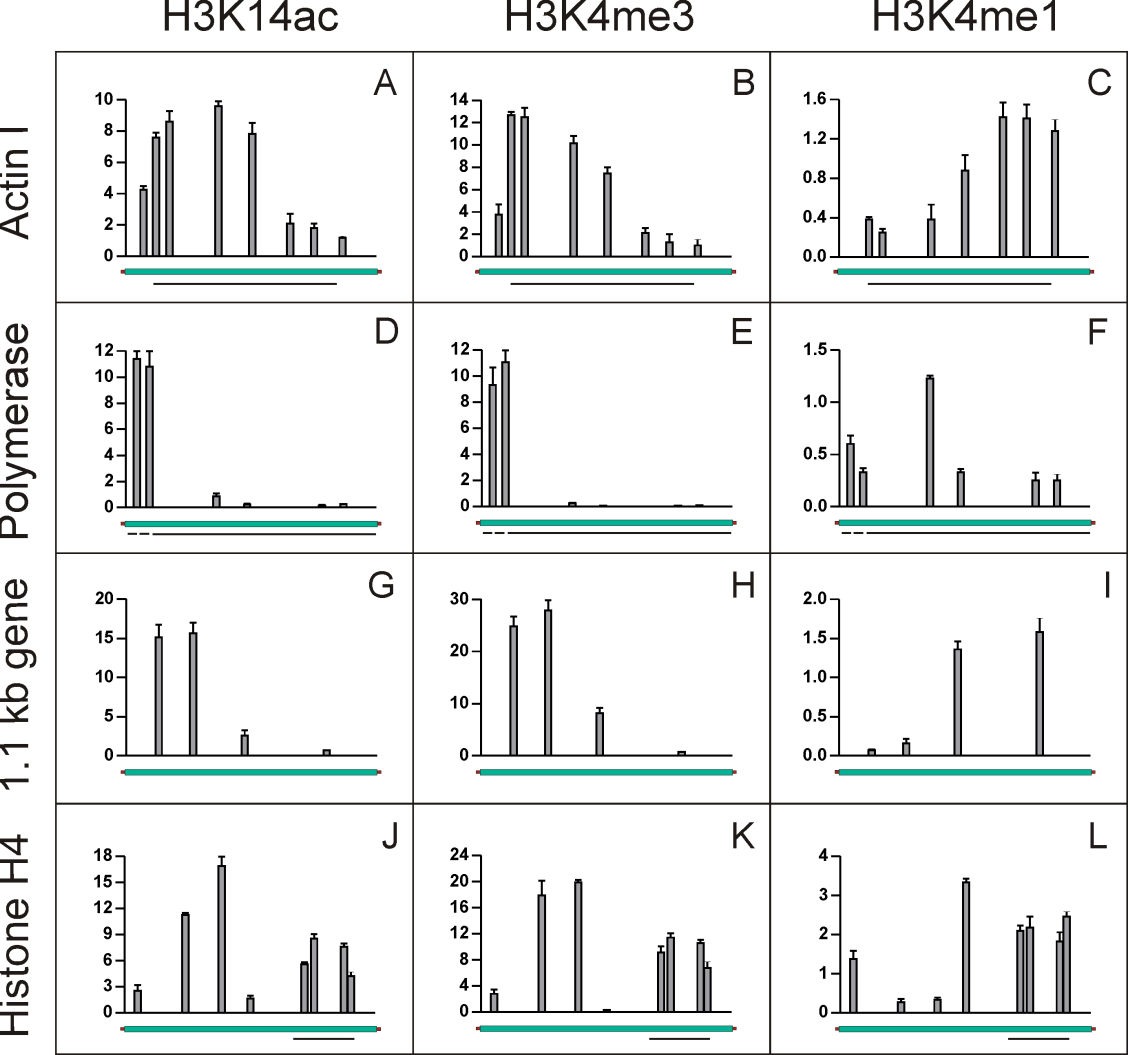
**H3K14ac, H3K4me3 and H3K4me1 distribution on actively transcribed macronuclear nanochromosomes**. Nanochromosomes encoding actin I (A-C), DNA polymerase alpha (D-F), 1.1 kb gene (G-I) and histone H4 (J-L) were examined. X-axis shows total length of gene, Y-axis shows percent of input. Data shown are derived from three individual ChIP experiments, error bars represent SE.

To identify patterns of PTMs on nanochromosomes encoding actively transcribed genes, chromatin was digested with micrococcus nuclease to yield mononucleosomes. Chromatin immunoprecipitation (ChIP) experiments and subsequent quantitative real-time PCR (qRT-PCR) analyses revealed that on nanochromosomes with short flanking sequences, e.g. actin I and polymerase? alpha, H3K14ac accumulated at the 5'-ends of genes and decreased towards the 3'-end (Figure 2). On the actin I nanochromosome the level of acetylated H3K14 increased steadily in the first half of the nanochromosome before declining in the second half, reaching the minimum at the 3'-end (Figure 2A). Interestingly, the amount of H3K14ac in the 5'-flanking region was lower than at the start of the coding sequence. The relative amount of H3K14ac in the polymerase alpha nanochromosome was very high at the 5'-end, corresponding to the start of the coding region and decreased significantly within the following 2 kb (Figure 2D). The distribution of H3K14ac on the 1.1 kb nanochromosome (Figure 2G) was very similar to that seen on the actin I (Figure 2A) and polymerase alpha nanochromosome (Figure 2D). In contrast to the data described above results obtained from the histone H4 nanochromosome differed remarkably. Whereas only one peak was detectable for nanochromosomes with short flanking sequences, we found two peaks of H3K14ac within the histone H4 nanochromosome. A first accumulation of H4K14ac was observed in the 5'-flanking region, followed by a second peak within the coding region (Figure 2J). We repeated these chromatin immunoprecipitation experiments using a combined antibody directed against H3K9/14ac. The results obtained were entirely consistent with those for the H3K14ac antibody (see Additional file 2A-D). Results obtained from ChIP analyses for trimethylated H3K4 were very similar to those obtained for H3K14ac. Again the examined nanochromosomes with short flanking untranslated regions, actin I (Figure 2B) and polymerase alpha (Figure 2E) showed an accumulation of H3K4me3 at the 5'-ends close to the start of the coding region and a significantly lower amount at the 3'-ends. A similar pattern was observed for the 1.1 kb nanochromosome (Figure 2H). In contrast, as for H3K14ac, two peaks of H3K4me3 were detected on the histone H4 nanochromosome, one in the 5'-flanking sequence and the other in the coding region (Figure 2K). Data obtained from ChIP and qRT-PCR analyses of H3K4me1 were less consistent compared to the data described for H3K14ac and H3K4me3. In the actin I nanochromosome the distribution of H3K4me1 seemed to mirror image the H3K4me3 distribution (Figure 2C). The level of H3K4me1 was low at the 5'-end and continuously increased towards the 3'-end reaching its highest concentration near the end of the coding region. The 1.1 kb gene exhibited a very similar distribution (Figure 2I). In contrast, the distribution of H3K4me1 on the polymerase alpha nanochromosome showed low amounts at both 5'-end and 3'-end of the coding region while the maximum level of H3K4me1 was found to reside in the middle of the gene (Figure 2F). H3K4me1 distribution in the histone H4 nanochromosome was different again in that its level was low at the 5'-end of the nanochromosome but peaked in the 5'-flanking sequence close to the coding region. Within the coding region this modification was almost evenly distributed (Figure 2L).

### H3K14ac and H3K4me3 do not associate with 5'-ends of silenced genes

**Figure 3:**
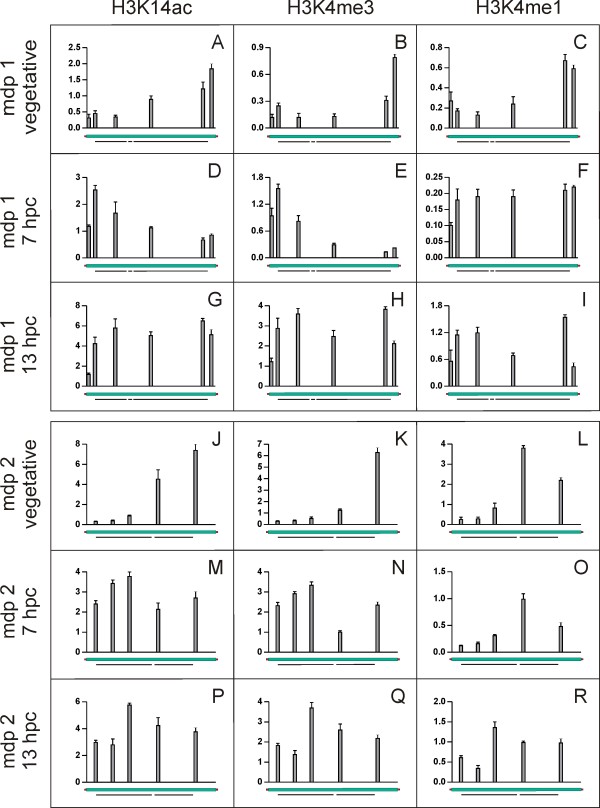
**Patterns of H3K14ac, H3K4me3 and H3K4me1 on silenced macronuclear nanochromosomes during vegetative growth and upon activation during conjugation**. The nanochromosome encoding mdp1 was examined during vegetative growth (A-C), 7 hpc (D-F) and 13 hpc (G-I). The nanochromosome encoding mdp2 was examined during vegetative growth (J-L), 7 hpc (M-O) and 13 hpc (P-R). X-axis shows total length of gene, Y-axis shows percent of input. Data shown are derived from three individual ChIP experiments, error bars represent SE.

To identify the PTM pattern on transcriptionally silent genes, ChIP and qRT-PCR experiments were performed using the same antibodies as described above. As shown in Figure 3A-C and Figure 3J-L the distribution of histone modifications differed significantly from those on actively transcribed genes (Figure 2). We found inactive genes to also associate with PTMs typical for open and permissive chromatin, although to a significantly lower amount, an observation also described in other organisms. However, not only the quantity differed but, more surprisingly, the PTM distribution also showed remarkable qualitative differences. While in active genes H3K14ac accumulated at the 5'-end of the nanochromosome, the relative amount of this modification was considerably higher at the 3'-end than at the 5'-end of the mdp1 (Figure 3A) and the mdp2 (Figure 3J) nanochromosomes. The amount of H3K14ac steadily increased over the nanochromosome to reach its maximum in the 3'-flanking region. Essentially the same was true for the distribution of H3K4me3 (Figure 3B, 3K). In contrast, the distribution of H3K4me1 in untranscribed nanochromosomes partly resembled that observed on actively transcribed genes. The level of monomethylated H3K4 was low at the 5'-end and increased towards the 3'-end. In both nanochromosomes the highest amount of H3K4me1 was found towards the 3'-end of the coding region (Figure 3C, 3L).

### H3K14ac and H3K4me3 are relocated upon gene activation

Since we observed significant differences in the PTM patterns in genes either actively transcribed or repressed during vegetative growth it seemed of considerable interest to analyse the PTM patterns upon gene activation. Mdp1 and mdp2 are not expressed during vegetative growth but mRNAs of both genes can be detected 6-8 hours after the initiation of conjugation [10]. We therefore isolated macronuclear chromatin from conjugating cells at 7 hpc (hours post conjugation) and performed ChIP and qRT-PCR analyses. The distribution of PTMs observed on these genes after activation of gene expression differed significantly from those in a silenced state. Upon activation, the pattern of H3K14ac and H3K4me3 was inverted compared to their silent status. On mdp1 the highest levels of acetylated H3K14 were found at the 5'-end at the beginning of the coding region and its concentration decreased towards the 3'-end (Figure 3D), reminiscent of the pattern on transcribed genes during vegetative growth. A similar observation was made for mdp2 (Figure 3M). These ChIP experiments were repeated using a combined antibody directed against H3K9/14ac. The results obtained were entirely consistent with those for the H3K14ac antibody (see Additional file 3A-F). The distribution of H3K4me3 (Figure 3E, 3N) was similar to that of H3K14ac. ChIP data obtained for H3K4me1 were not as consistent as the other modifications. While on mdp2 no change in H3K4me1 distribution was detected (Figure 3O) a significant redistribution on mdp1 was observed (Figure 3F). In the repressed mdp1 gene H3K4me1 was predominantly found at the 3'-end but spread after activation almost over the entire nanochromosome. As a control, we analysed genes expressed during both, vegetative growth and conjugation. None of the PTM patterns on the actin I (see Additional file 4A-C) or the polymerase alpha nanochromosome (see Additional file 4D-F) changed during conjugation, indicating that relocation of PTMs is induced only upon gene activation. It has been reported that in the course of conjugation overall gene expression increases at 6-8 hpc [18] before it declines and reaches a low level. Therefore, we also analysed PTM patterns on the mdp1 and mdp2 nanochromosomes at a later time point after conjugation (13 hpc) when the overall transcription rate had already decreased. At this point PTM patterns were not as clearly structured as described for 7 hpc. H3K14ac, H3K4me3 and H3K4me1 were more or less evenly distributed over the entire nanochromosomes of both mdp1 and mdp2 (Figure 3G-I, 3P-R), suggesting that we observe a transitional stage in which transcription rate already becomes reduced.

### During nuclear development genes exhibit PTM patterns different from those of actively transcribed genes

**Figure 4:**
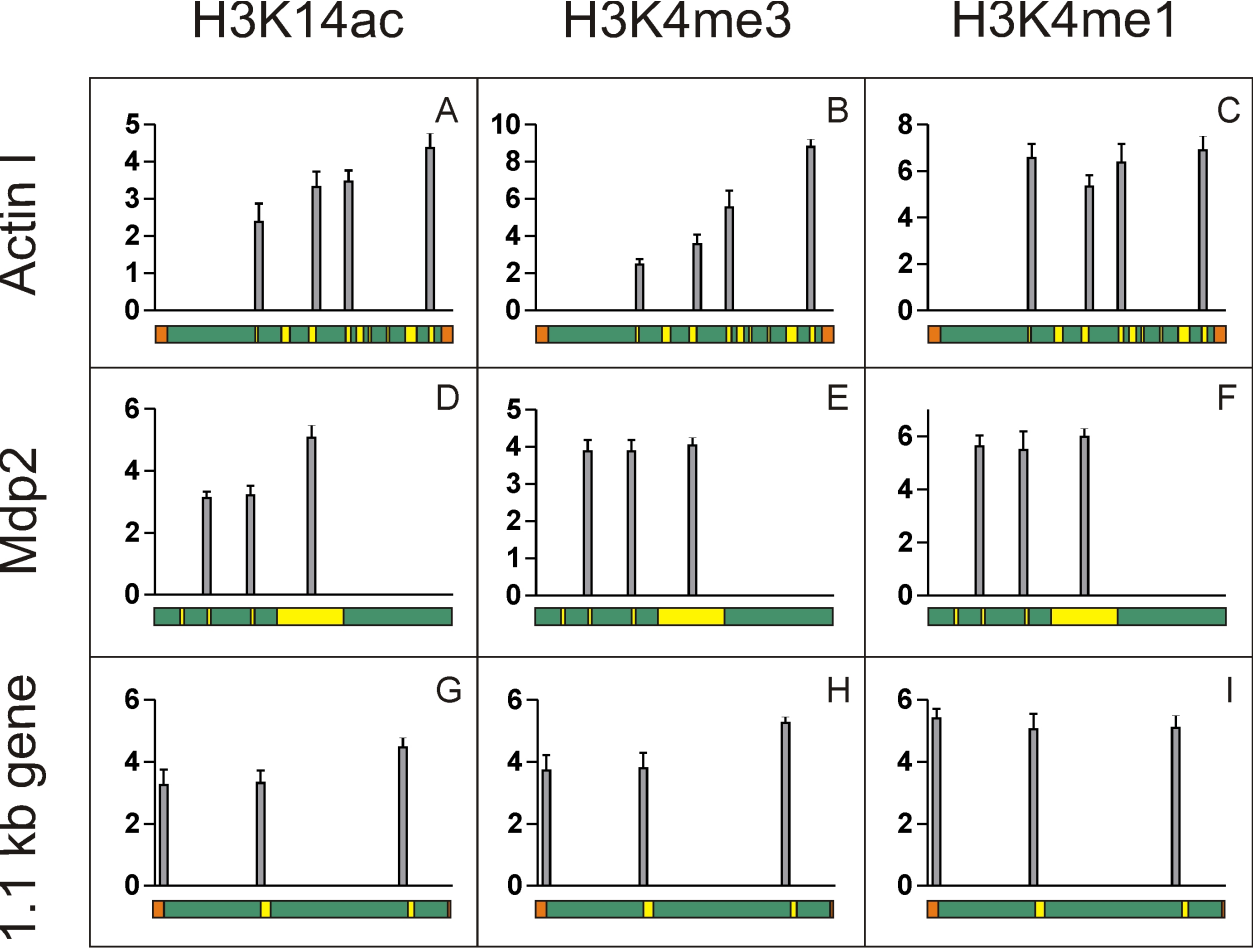
**Patterns of H3K14ac, H3K4me3 and H3K4me1 on macronuclear-destined sequences during macronuclear development**. Sequences encoding actin I (A-C), mdp2 (D-F) and the 1.1 kb gene (G-I) were examined. X-axis shows total length of gene, Y-axis shows percent of input. Data shown are derived from three individual ChIP experiments, error bars represent SE.

Chromatin was isolated from macronuclear anlagen (30 hpc) and the distribution of 3 PTMs on macronuclear-specific sequences was analysed by ChIP and qRT-PCR. To avoid amplification of macronuclear contaminations in ChIP with antibodies directed against H3K14ac, H3K4me3 and H3K4me1 anlagen-specific primers were used for qRT-PCR analyses (Figure 1C). At this stage of anlagen differentiation the patterns of H3K14ac and H3K4me3 unambiguously differed from those in actively transcribed genes during vegetative growth. In the actin I gene the level of H3K14ac was low near the 5'-end and steadily increased towards the 3'-end of the gene (Figure 4A). The same was true for H3K4me3 (Figure 4B). H3K4me1 in contrast was evenly distributed over the gene (Figure 4C). Unfortunately, due to the lack of suitable primer combinations, the very 5'-end of the actin I sequence could not be analysed. With the exception of H3K4me3 which is evenly distributed over the sequence, results from the analyses of PTM distributions on the mdp2 (Figure 4D-F) gene resemble those from actin I (Figure 4A-C). The data obtained from the 1.1 kb nanochromosome also suggest a similar distribution of single PTMs on this gene (Figure 4G-I). However, although few anlagen-specific sequences could be found within the 1.1 kb sequence, many of them were not suited for qRT-PCR. Therefore, PCR fragment c (Figure 1C) had to be amplified using macronucleus-specific primers so that simultaneous amplification of contaminating macronuclear DNA can not be excluded. Similar analyses of other genes were technically impossible due to micronuclear complexity of the polymerase alpha gene [19], the micronuclear simplicity of the histone H4 gene and limited knowledge of micronuclear sequence of mdp1.

## Discussion

Post-translational histone modifications influence chromatin structure and are therefore involved in the regulation of gene activity [1]. The observation that some of these PTMs are not evenly distributed over a gene but accumulate at the 3'- or 5'-end in several organisms suggests that also local chromatin changes are required for the modulation of gene expression [3,4,17]. We decided to exploit the simplified ciliate model system to address the question of PTM dynamics during gene activation and nuclear differentiation. In the macronucleus the DNA is organized into nanochromosomes, each encoding a gene and all sequences required for expression and replication, hence representing functional units independent of a large genomic context. Since some genes are activated only in the course of sexual reproduction, changes in PTM pattern can easily be analysed after their activation. In addition, the morphological events during macronucleus differentiation are well defined and specific stages of this differentiating nucleus can be isolated in large quantities allowing the characterisation of PTM pattern on defined genes during differentiation. The histone modifications generally associated with active chromatin considered in this report were chosen because it has been shown before that they are present both in the vegetative and developing macronucleus [11].

We initially analysed the PTM pattern on actively transcribed nanochromosomes and found a distribution similar to that described in other organisms [4,5,15,17]. H3K14ac and H3K4me3 predominantly accumulated near the transcriptional start site at 5'-end of genes (Figure 2). As we were able to detect these PTMs within the immediate neighborhood of the promoter region we can exclude the eviction of nucleosomes from these sites as it has been described in yeast [5]. The maximum amount of H3K4me1 was found to reside near the middle or the 3'-end of the coding region (Figure 2). Only the nanochromosome encoding the histone H4 gene with its extraordinary long 5'-flanking region showed a different pattern. H3K14ac and H3K4me3 accumulated both in the 5'-flanking regions and in the open reading frame (Figure 2). The reason for this distribution is not clear. Since we could not find an ORF in the 5'-region it may well be that a regulatory element relevant for expression is contained within this sequence. It has been described before that e.g. enhancers carry specific PTM patterns [15,20].

The PTM patterns on the silenced genes mdp1 and mdp2 differed remarkably from those of actively transcribed genes. As described in other organisms PTMs characteristic for open chromatin decorate these inactive genes only in a low amount. Surprisingly however, H3K14ac and H3K4me3 were found to predominantly accumulate not at the 5'-end but at the 3'-end of the nanochromosomes while the distribution of H3K4me1 remained unchanged (Figure 3). It has been described that in bivalent domains the permissive marker H3K4me3 accumulates together with the repressive marker H3K27me3 [6,7]. By this mechanism inactive genes in embryonic stem cells [21] and CD4^+ ^T cells [8,22] are kept poised for activation when needed upon differentiation. Importantly, here we demonstrate that not only the sole existence but the distribution of PTMs on a gene correlates with its transcriptional activity.

While in other organisms no qualitative but only quantitative differences in PTMs were observed when comparing genes of different transcriptional status [5,15-17] we observe a significant relocation of PTMs typical for permissive chromatin on the nanochromosomes encoding mdp1 and mdp2 upon activation (Figure 3). Histones at the 5'-end become acetylated and methylated *de novo *while these modifications are most likely removed from histones at the 3'-end of genes. The patterns of H3K14ac and H3K4me3 resemble those of genes actively transcribed during vegetative growth. This implies that a mechanism must exist that directs the remodelling of PTMs during gene activation.

It has been described in *Stylonychia *that immediately following the fusion of the gametic nuclei a global *de novo *acetylation of histones takes place [11] which is reminiscent of the early embryonic cell nuclei in metazoa [23]. Only later repressive histone modifications become introduced. It has been described that in *Stylonychia *macronuclear-specific DNA sequences to be retained in the vegetative macronucleus stay associated with permissive histone modifications while all sequences to be eliminated during further differentiation are marked by repressive histone modifications, a mechanism reminiscent to the silencing of genes during metazoan development [23]. Our analyses now reveal that the distribution of active PTMs in the differentiating macronucleus does not resemble that of actively transcribed genes during vegetative growth, which suggests a silenced transcriptional status during nuclear development (Figure 4). This result is consistent with the view that, although these sequences have to be associated with permissive histone modifications in order to become processed into vegetative genes, they are not expressed during macronuclear differentiation but have to be activated upon termination of the differentiation process.

Taking all our observations together, we can propose a mechanistic model how PTM patterns correlate with genetic activity and the relevance of this pattern for genes during a differentiation process. In expressed genes a typical distribution of PTMs over the gene is observed as already described in other eukaryotes [3-5,15,17]. Silenced genes do also associate with PTMs characteristic for open chromatin, but only to a low amount. In contrast to actively transcribed genes, the PTM pattern on silenced genes is almost a mirror image of that of active genes. While in expressed genes H3K14ac and H3K4me3 always accumulate at the start site of transcription, these modifications are found preferentially in the 3'-region of silenced genes. In the course of gene activation, a quantitative and qualitative change in PTM patterns is observed and PTMs are relocated on the activated gene. In a process most probably involving deacetylases and demethylases as well as acetyltransferases and methyltransferases distinct PTMs are removed from the 3'-end while histones near the TSS gain such modifications. During early differentiation genes are marked by permissive histone modifications. The differing distribution of permissive markers serves to prevent DNA sequences from being transcribed as well as to prevent them from being eliminated during differentiation. It will be of major interest to perform similar analyses in metazoan organisms and the experiments described may serve as guidelines for such investigations.

## Conclusion

So far, only a quantitative change of specific PTMs has been observed during gene activation in eukaryotic cells but the mechanism directing these is only beginning to be understood. We demonstrate in the simplified model system ciliate that gene activation correlates not only with a quantitative but also a qualitative change in specific PTMs. This process has to be regulated by concerted demethylation and deacetylation followed by *de novo *methylation and acetylation. Furthermore, we show the relevance of PTMs in a differentiating nucleus. Results described in this paper may serve as guidelines to perform similar analyses in higher eukaryotes.

## Methods

### Growth of *Stylonychia lemnae*

Growth of *Stylonychia lemnae *was performed as described elsewhere [18]. For mating, cells of two different mating types were mixed and allowed to conjugate [18].

### Native chromatin preparation from vegetative macronuclei, macronuclei from conjugating cells and macronuclear anlagen

To all buffers 1 mM PMSF or protease inhibitor cocktail (Roche Diagnostics) was added to avoid protein degradation as well as 5 mM sodium butyrate to avoid histone deacetylation. Nuclei were isolated as described elsewhere [18], washed once with cold PBS and resuspended in 1 ml micrococcus nuclease (MNase) digestion buffer (50 mM NaCl, 20 mM Tris-HCl (pH 7.5), 3 mM MgCl_2_, 1 mM CaCl_2_). Nuclei were digested with 0.25 units MNase per μg DNA for 10 min at 37°C. Reaction was stopped with 0.5 mM EDTA. Nuclei were placed on ice for 1 h and homogenized to release mononucleosomes. The nuclear suspension was centrifuged for 5 min at 2500 × g and the supernatant was transferred to a new reaction tube. The pelleted nuclei were resuspended in 600 μl MNase wash buffer (10 mM Tris-HCl (pH 7.5), 250 μM EDTA) and kept on ice for 1 h with repeated homogenization. The last step was repeated once. Finally, the combined supernatants were concentrated in VivaSpin4 concentrators (Sartorius). For the isolation of macronuclear chromatin from cells after conjugation, *Stylonychia *clones of different mating types were mixed and macronuclei were isolated 7 and 13 hours, respectively, after the start of conjugation. Chromatin from macronuclear anlagen was isolated 30 hours after the start of conjugation. All chromatin preparations used for ChIP analyses were analysed by agarose gel electrophoresis.

### Chromatin immunoprecipitation (ChIP)

Protease inhibitor cocktail was added to all buffers and when appropriate 5 mM sodium butyrate was added to avoid histone deacetylation. The lysate containing mononucleosomes was cleared by centrifugation (14,000 × g, 5 min, 4°C). For each ChIP 0.5 OD_260 _chromatin corresponding to 25 μg DNA were diluted in 500 μl ChIP incubation buffer (50 mM NaCl, 20 mM Tris-HCl (pH 7.5), 5 mM EDTA), and pre-cleared with Sepharose A beads for 2 h at 4°C. Beads were pelleted by centrifugation (150 × g, 5 min, 4°C), and the supernatant was transferred to a new reaction tube. At this step one aliquot of pre-cleared chromatin was kept as 'input DNA' for future qRT-PCR analysis. Sepharose A beads were blocked with BSA and sheared salmon sperm DNA for 2 h at 4°C in buffer A (50 mM NaCl, 50 mM Tris-HCl (pH 7.5), 10 mM EDTA), centrifuged (150 × g, 5 min, 4°C) and resuspended in buffer A. Immunoprecipitation was performed overnight using 3 μg of specific antibody and blocked Sepharose A beads at 4°C at gentle rotation. Antibodies used were anti-H3K14ac (Upstate, up07-353), anti-H3K9/14ac (Santa Cruz, sc-8655-R), anti-H3K4me3 (Abcam, ab8580) and anti-H3K4me1 (Abcam, ab8895). Immunocomplexes were washed under gentle rotation at 4°C for 10 min as follows: once with buffer A, twice with buffer B (100 mM NaCl, 50 mM Tris-HCl (pH 7.5), 10 mM EDTA) and twice with buffer C (150 mM NaCl, 50 mM Tris-HCl (pH 7.5), 10 mM EDTA). To elute the precipitated chromatin, immunocomplexes were incubated with elution buffer (50 mM NaCl, 20 mM Tris-HCl (pH 7.5), 5 mM EDTA, 1% SDS) for 30 min at 65°C on a shaker. Proteinase K was added to the eluate and incubated at 65°C for 2 h. DNA was purified using phenol-chloroform extraction and ethanol precipitation. ChIP experiments were repeated three times.

### Quantitative real-time PCR

Quantitative real-time PCR (qRT-PCR) analyses were performed as described before [11]. Primers used for qRT-PCR analyses are listed in Additional files 5 and 6, their positions are shown in Figure 1.

## Abbreviations

PTM: Post-translational histone modification; mdp: Macronuclear development protein; hpc: Hours post conjugation; H3K14ac: Histone H3 acetylated at lysine 14; H3K4me3: Histone H3 trimethylated at lysine 4; H3K4me1: Histone H3 monomethylated at lysine 4; qRT-PCR: Quantitative Real-Time PCR

## Authors' contributions

KSH performed ChIP and qRT-PCR analyses of actin I, histone H4, mdp1 and mdp2 in the vegetative macronucleus, ChIP and qRT-PCR analyses in the macronucleus during sexual reproduction, ChIP and qRT-PCR analyses in the differentiating macronucleus, participated in the design and coordination of the study and in drafting the manuscript. SEW performed the ChIP and qRT-PCR analyses of polymerase alpha and the 1.1 kb gene in the vegetative macronucleus. HJL participated in the design and coordination of the study and drafted the manuscript. All authors read and approved the final manuscript.

## Supplementary Material

Additional file 1 **Characterisation of genes analysed in this study**. Indicated are total size, size of 5'- and 3'-flanking regions and number of introns. Click here for file

Additional file 2 **Pattern of H3K9/14ac on macronuclear nanochromosomes**. Distribution of H3K9/14ac is shown in actin I (A), DNA polymerase alpha (B), the 1.1 kb gene (C) and histone H4 (D). X-axis shows total length of gene, Y-axis shows percent of input. Data shown are derived from three individual ChIP experiments, error bars represent SE. Click here for file

Additional file 3 **Pattern of H3K9/14ac on silenced macronuclear nanochromosomes during vegetative growth and upon activation during conjugation**. The nanochromosome encoding mdp1 was examined during vegetative growth (A), 7 hpc (B) and 13 hpc (C). The nanochromosome encoding mdp2 was examined during vegetative growth (D), 7 hpc (E) and 13 hpc (F). X-axis shows total length of gene, Y-axis shows percent of input. Data shown are derived from three individual ChIP experiments, error bars represent SE. Click here for file

Additional file 4 **Distribution of H3K14ac, H3K4me3 and H3K4me1 on actively transcribed macronuclear nanochromosomes during conjugation**. The nanochromosomes encoding actin I (A-C) and polymerase alpha (D-F) were examined 7 hpc. X-axis shows total length of gene, Y-axis shows percent of input. Data shown are derived from three individual ChIP experiments, error bars represent SE. Click here for file

Additional file 5 **Table of primers used for qRT-PCR analyses of macronuclear nanochromosomes**. Primer names correlate with numbers of PCR fragments indicated in Figure 1A and 1B. Click here for file

Additional file 6 **Table of primers used for qRT-PCR analyses of MDSs in the differentiating macronucleus**. Primer names correlate with letters of PCR fragments indicated in Figure 1C. Click here for file
