# Supplementary figures and images for: Histone modifications are specifically relocated during gene activation and nuclear differentiation
Source: BMC Genomics. 2009 Nov 24;10:554. doi: 10.1186/1471-2164-10-554 (PMC2787535; doi:10.1186/1471-2164-10-554)

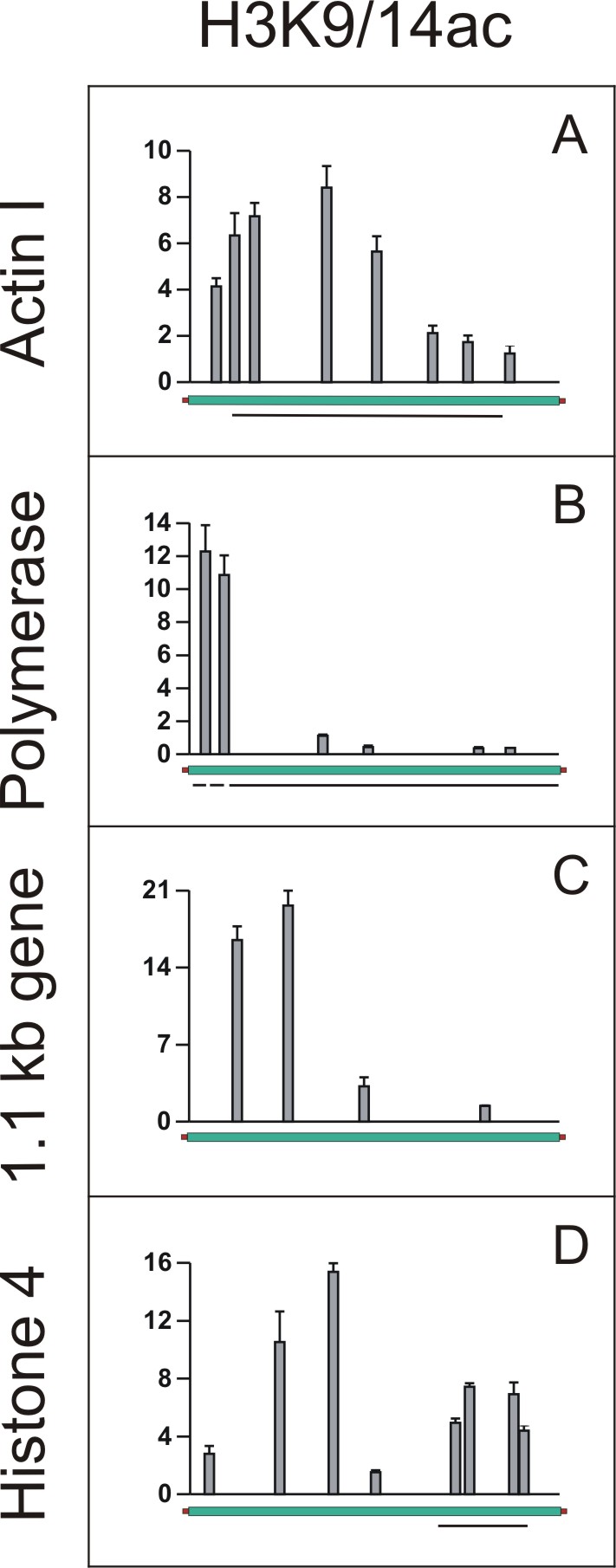

Supplement: Additional file 2 — Pattern of H3K9/14ac on macronuclear nanochromosomes. Distribution of H3K9/14ac is shown in actin I (A), DNA polymerase alpha (B), the 1.1 kb gene (C) and histone H4 (D). X-axis shows total length of gene, Y-axis shows percent of input. Data shown are derived from three individual ChIP experiments, error bars represent SE. [file 1471-2164-10-554-S2.JPEG]

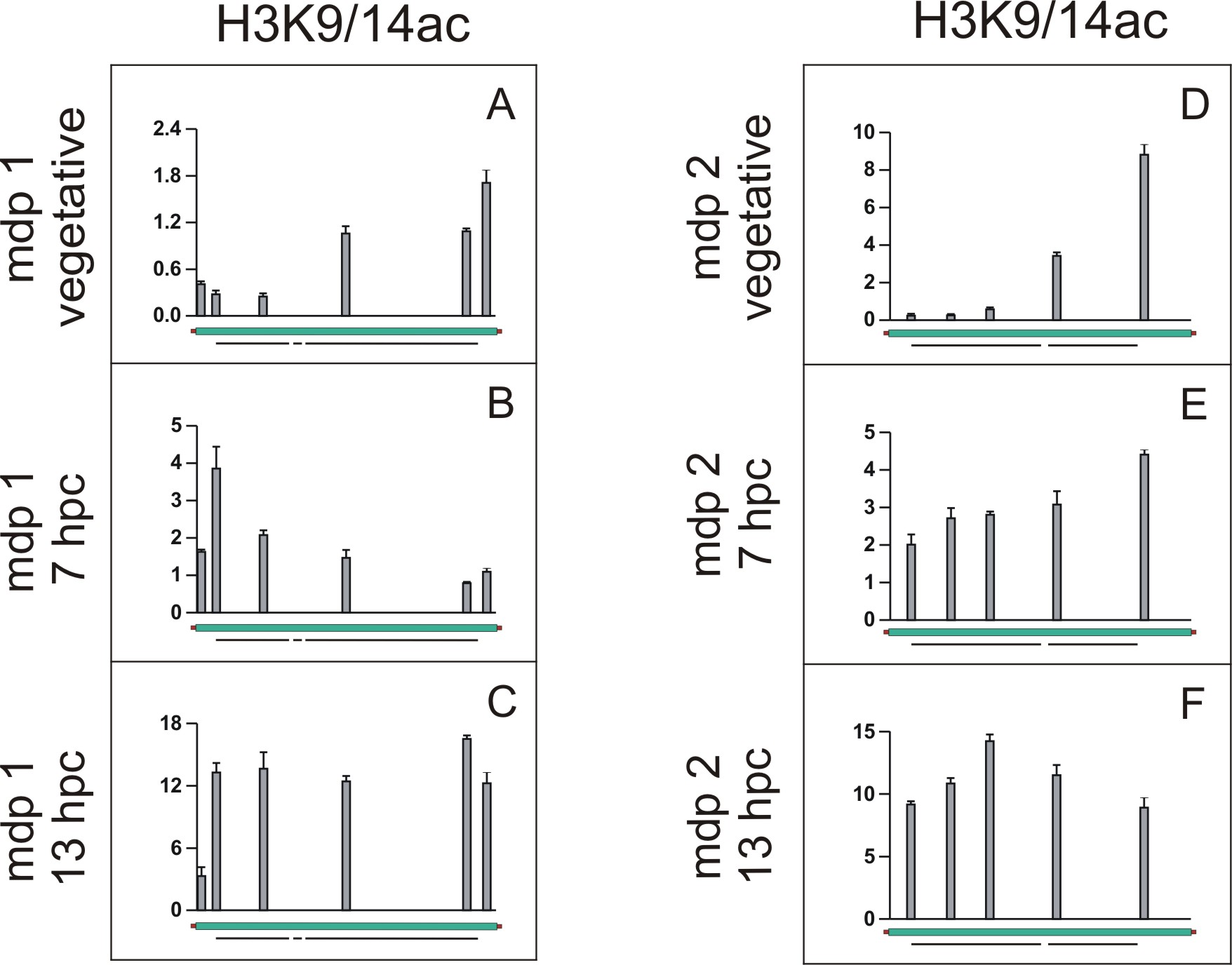

Supplement: Additional file 3 — Pattern of H3K9/14ac on silenced macronuclear nanochromosomes during vegetative growth and upon activation during conjugation. The nanochromosome encoding mdp1 was examined during vegetative growth (A), 7 hpc (B) and 13 hpc (C). The nanochromosome encoding mdp2 was examined during vegetative growth (D), 7 hpc (E) and 13 hpc (F). X-axis shows total length of gene, Y-axis shows percent of input. Data shown are derived from three individual ChIP experiments, error bars represent SE. [file 1471-2164-10-554-S3.JPEG]

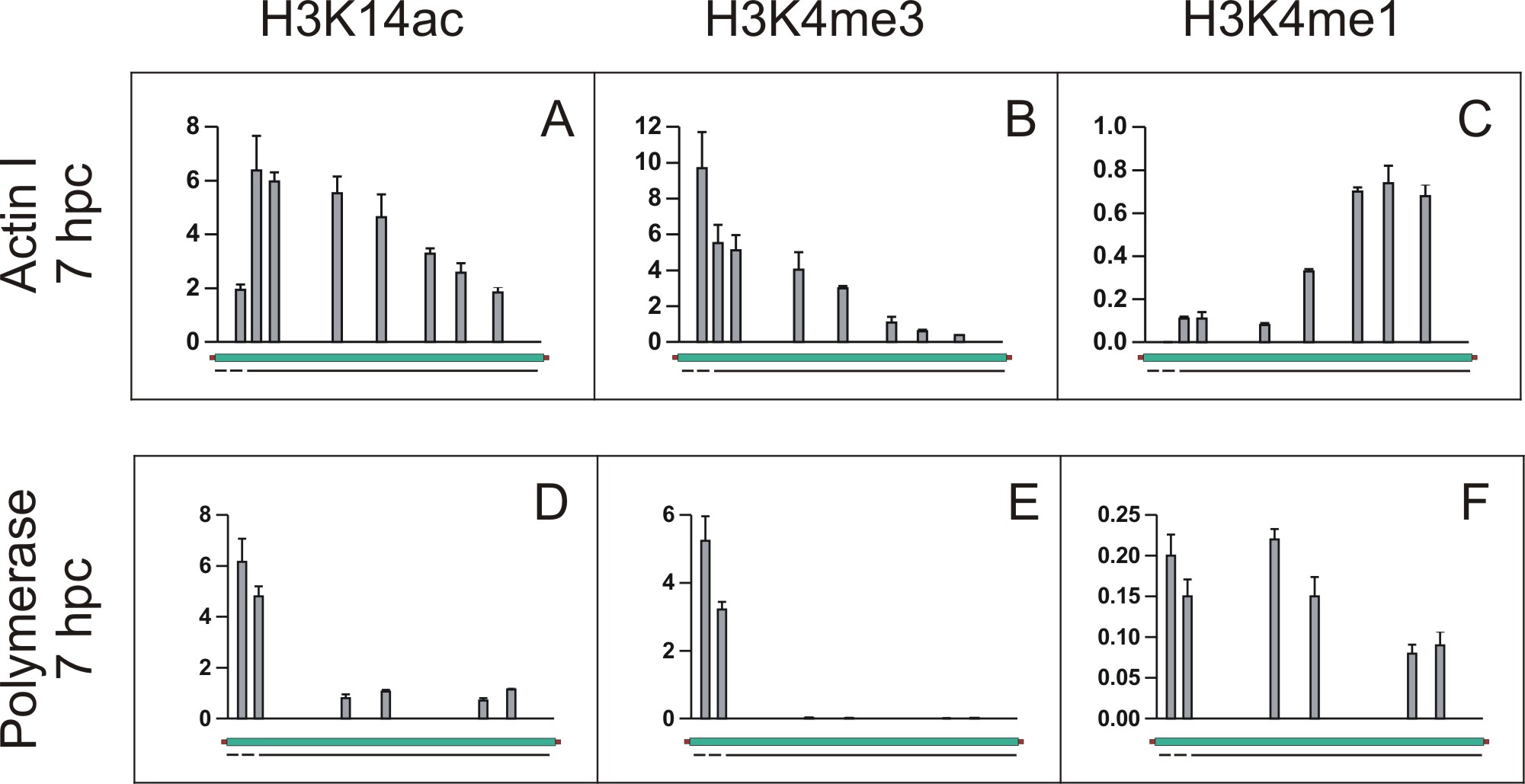

Supplement: Additional file 4 — Distribution of H3K14ac, H3K4me3 and H3K4me1 on actively transcribed macronuclear nanochromosomes during conjugation. The nanochromosomes encoding actin I (A-C) and polymerase alpha (D-F) were examined 7 hpc. X-axis shows total length of gene, Y-axis shows percent of input. Data shown are derived from three individual ChIP experiments, error bars represent SE. [file 1471-2164-10-554-S4.JPEG]
